# Intracellular targeting with engineered proteins

**DOI:** 10.12688/f1000research.8915.1

**Published:** 2016-08-10

**Authors:** Shane Miersch, Sachdev S. Sidhu

**Affiliations:** 1 Banting and Best Department of Medical Research, Donnelly Centre for Cellular and Biomolecular Research, University of Toronto, Toronto, Ontario, Canada

**Keywords:** proteins, intracellular targeting, protein interactions

## Abstract

If the isolation, production, and clinical use of insulin marked the inception of the age of biologics as therapeutics, the convergence of molecular biology and combinatorial engineering techniques marked its coming of age. The first wave of recombinant protein-based drugs in the 1980s demonstrated emphatically that proteins could be engineered, formulated, and employed for clinical advantage. Yet despite the successes of protein-based drugs such as antibodies, enzymes, and cytokines, the druggable target space for biologics is currently restricted to targets outside the cell. Insofar as estimates place the number of proteins either secreted or with extracellular domains in the range of 8000 to 9000, this represents only one-third of the proteome and circumscribes the pathways that can be targeted for therapeutic intervention. Clearly, a major objective for this field to reach maturity is to access, interrogate, and modulate the majority of proteins found inside the cell. However, owing to the large size, complex architecture, and general cellular impermeability of existing protein-based drugs, this poses a daunting challenge. In recent years, though, advances on the two related fronts of protein engineering and drug delivery are beginning to bring this goal within reach. First, prompted by the restrictions that limit the applicability of antibodies, intense efforts have been applied to identifying and engineering smaller alternative protein scaffolds for the modulation of intracellular targets. In parallel, innovative solutions for delivering proteins to the intracellular space while maintaining their stability and functional activity have begun to yield successes. This review provides an overview of bioactive intrabodies and alternative protein scaffolds amenable to engineering for intracellular targeting and also outlines advances in protein engineering and formulation for delivery of functional proteins to the interior of the cell to achieve therapeutic action.

## Introduction

Proteins transmit signals that mediate cellular function and dysfunction in part via assembly into complexes with other proteins through binding interfaces responsible for transient or enduring interactions. These sites of protein-protein interaction represent a class of drug targets that allow cell signals to be modulated. Interaction interfaces have been probed extensively and have been revealed to assume both gross planar or curved topologies with complex localized surfaces ^1^. Their areas tend to range from about 1000 to 2000 Å ^2^ (with some much larger) and tend to possess a higher proportion of hydrophobic residues than the exposed protein surface ^2– 4^. As a result of the large surfaces and number of weak bonds that collectively give rise to affinity between proteins, small molecules have largely been less effective in disrupting protein complexes and tend rather to target hydrophobic pockets or clefts ^5^. Estimates of the number of proteins that possess functional dependence on such structural features, or the small-molecule “druggable” portion of the proteome, suggest this to encompass only about 10% of the entire complement of human proteins ^6^. As a result, protein-protein interactions thought to be undruggable or high-risk targets using conventional small molecules, through herculean efforts, have rendered some interactions druggable ^7^. Nevertheless, owing to their small size and a relative paucity of interactions, targeting with small molecules can also lead to issues with specificity and off-target effects that undermine their pharmacological profile.

Alternately, owing to their increased size and propensity for electrostatic and geometric complementarity, protein-based drugs such as antibodies are generally better suited to disruption of the large, flatter but topologically complex surfaces that comprise sites of protein-protein interactions (although camelids and V _H_ domains may expand the scope of accessible targets because of their observed ability to bind surface clefts ^8, 9^) and are less prone to off-target effects. In the extracellular space, antibodies have proven to be a versatile means for targeting and modifying the activities of proteins. They have consequently altered the therapeutic landscape and now constitute the dominant class of new pharmaceuticals ^10^. Advances in display technologies, means of generating combinatorial libraries, and an expanding repertoire of non-antibody scaffolds continue to revolutionize the field of protein engineering and move towards the clinic in support of a burgeoning biologics-based pharmaceutical industry ^11, 12^. Currently, a broad variety of scaffold topologies are under development for drug applications and ensure that complementary binding agents should be available for the similarly broad array of target surfaces.

The delivery of protein-based drugs directly to the cell interior represents perhaps the last major hurdle in accessing the vast array of protein-protein interactions inside the cell that are not currently amenable to targeting by small-molecule drugs. Historically, protein modulation inside the cell has been accomplished indirectly by expressing ectopic proteins encoded by DNA delivered into the cell or through disruption of protein expression by using RNA interference. Despite the utility of these approaches, a host of drawbacks largely preclude their use as a means of therapeutic intervention (discussed below). Alternately, direct protein transduction could replace dysfunctional proteins without the need for expression, enable the use of non-canonical amino acid residues in therapeutics, and make possible direct modulation of target proteins, post-translationally modified proteins, and conformational variants that, though potentially targeted by genetic methods (that is, via direct expression from encoding constructs), could further enhance control over intracellular therapeutic concentrations.

## Proteins engineered to target and modulate intracellular proteins

There are many examples in which intracellular protein function (that is, nuclear, cytoplasmic, mitochondrial, lysosomal, endoplasmic reticulum, and so on) is at the root of disorders, including oncogenic, autoimmune, and degenerative diseases ^13– 15^. These proteins include modulators of gene expression, cell cycle progression, protein folding and apoptosis, novel chimeric products of gene translocations, oncogenic proteins, and many more, and they offer a rich and vast trove of potential targets for therapeutic intervention.

Antibodies have been highly effective in targeting cell surface proteins involved in disease development. Though it is generally believed that their large size, complex architecture, and structural reliance on disulfide bonds preclude intracellular application, a number of examples of both *in situ*-expressed ^16^ and exogenously supplied whole antibodies ^15– 20^ shown to maintain functional intracellular activity exist in the literature. Attempts to use smaller, less complex binding proteins such as antigen-binding fragments (Fabs) and single-chain variable fragments (scFvs) for intracellular application have similarly shown success in their ability to bind and modulate cytoplasmic protein function ^18– 21^. However, they can be susceptible to disulfide bond reduction, and aggregation of *in situ-*expressed antibody fragments is a common occurrence in the reducing environment of the cytoplasm ^23– 26^. To circumvent these issues, investigators have developed novel engineering and selection strategies that seek to (a) identify minimal binding frameworks ^22, 23^, (b) increase stability ^24– 27^, (c) eliminate reliance on disulfide bonds ^22, 23^, and (d) identify validated functional binding intrabodies ^28– 30^. These efforts have been complemented by additional studies confirming the delivery and retention of *in situ*-expressed intrabodies in specific subcellular compartments (nuclear, cytoplasmic, endoplasmic reticular, and peroxisomal) by fusing them to signal sequences to promote interaction with sequestered target proteins ^31– 33^. In addition, the growing number of proteins effectively targeted by intrabodies suggests ongoing interest in their future role as potential therapeutic agents for mitigation of disease ^33– 37^.

The number of novel, domain-sized proteins demonstrated as affinity agents that can be engineered for specificity is also growing. These scaffolds are finding increasing application because of their small size, lack of cysteines, and stable folding, and the wide array of topologies shown to be amenable to engineering ensures the availability of potentially complementary binding agents to virtually any target ^12, 38^. Most of these are currently under development for extracellular applications; however, several notable intracellular applications under development warrant discussion.

A general approach has recently been described to develop reagents, probes, and potential therapeutics against the enzymes of the ubiquitin system ^39^. Post-translational ubiquitination of proteins is a mode of signal transduction used pervasively inside the cell to regulate numerous critical cellular functions ^41, 42^. The large, solvent-accessible surface of ubiquitin that mediates low-affinity interactions with a variety of proteins was recognized as being amenable to engineering, and the surface was randomized in combinatorial libraries to isolate high-affinity variants with selectivity for enzymes of the ubiquitin cascade. In a series of studies, novel variants based on the ubiquitin scaffold were developed as tools to probe the various ubiquitinating (E1, E2, and E3) ligases, deubiquitinases (DUBs), and ubiquitin-binding domains ^39– 41^. By isolating a series of high-affinity ubiquitin variants (Ubvs) to numerous DUBs ^32^, HECT E3 ligases ^39, 41^, and SCF E3 ligases ^40^ that were capable of either antagonizing or agonizing the activity of its target both *in vitro* and in a cellular milieu, a path for addressing the knowledge lag on ubiquitination signaling versus other major pathways (for example, phosphorylation/dephosphorylation) has been established. To underscore the utility of Ubvs in probing enzyme cascades, investigators confirmed Ubv expression and interactions with intracellular targets by co-immunoprecipitation, observed expected changes in the ubiquitination and stability of ligase or DUB targets, and characterized downstream signals modulated by variants ^39– 41^. Taken together, these studies not only provide a foundation for continued inquiry into and characterization of ubiquitin signaling pathways but also lay the groundwork for therapeutic intervention in which dysfunction of ubiquitin cascades is at the root of disease.

Monobodies derived from the tenth type III domain of human fibronectin (FN3) have been widely used for a variety of extracellular applications ^42^, and some variants are under clinical evaluation ^43^. Their appeal stems in part from a lack of disulfide bonds and a stable structure resembling an immunoglobulin fold with six loops (three at either end of the molecule), five of which can be randomized without excessive destabilization. Selections against the SH3 domain of Fyn using phage-displayed combinatorial libraries based on the FN3 scaffold yielded a variant that binds to several family members (but not to closely related kinases) in a neutral fashion (that is, does not modify enzyme activity or interactions) but bears sensitivity to the conformational state of the target. Activation of Src is known to relieve intramolecular bonds within the SH3 domain that enables auto-phosphorylation. Though selected variants did not influence the kinase activity of Src, they did appear to exhibit selectivity for an active, open conformation in immunoprecipitation assays conducted in the presence or absence of the Src kinase activator ciglitazone. This enabled the use of fluorogenically tagged analogs of the binding variant to image SFK activation at the leading edges of cells as an increase in fluorescence upon target binding following microinjection into cells. This provides a prescient example that could illuminate the engineering of therapeutic biological agents that target disease-associated protein conformations to provide enhanced specificity ^44^.

Another valuable demonstration of the utility of non-antibody affinity scaffolds against intracellular proteins involved selective targeting of post-translational phosphor-modifications on the mitogen-activated protein kinase (MAPK) family member extracellular signal-regulated kinase 2 (ERK2). The designed ankyrin repeat protein (DARPin) scaffold was used to isolate binders to the phosphorylated and non-phosphorylated forms of ERK2 ^45^. The DARPin framework is composed of a series of repeating 3.5 kDa modules with both invariant framework regions and potential binding surface residues amenable to randomization. Four-module DARPins (14 kDa) targeting either of the two forms of ERK2 were used in a bioluminescence resonance energy transfer (BRET) assay to provide evidence of intracellular target binding. Serum stimulation of cells resulted in a loss of signal for the unphosphorylated ERK2 and an increase in signal for the phosphorylated form. Alternately, treatment with the ERK pathway inhibitor PD98509 resulted in an increase in signal for native ERK2 and a loss in the phosphor-signal despite no overall changes in ERK2 levels. Though the investigators did not explore the consequences of binding on downstream elements of the signaling cascade, these results suggest that DARPins could modify ERK2 signals in a manner that could offer therapeutic value given their role in inflammation, apoptosis, and oncogenic transformation.

**Figure 1.**
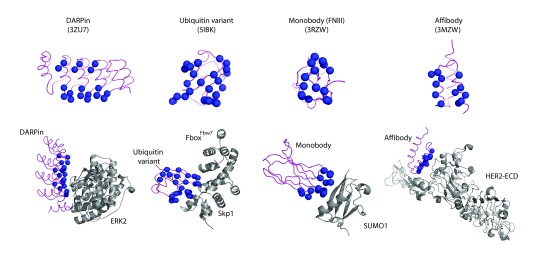
Alternative scaffolds used for the modulation of intracellular protein targets. The main chains of non-antibody scaffolds used to generate variants against intracellular targets are rendered in purple, and residues of the binding surface known to tolerate randomization are depicted as blue spheres. Structures in complex with intracellular target proteins (grey ribbons) are shown below. No structures exist for affibodies bound to intracellular targets and thus the structure of an affibody bound to the extracellular domain of HER2 is shown.

In addition to binders against a variety of extracellular targets, affibodies, based on a tri-helical bundle derived from the Z domain of *Staphylococcus aureus*, have been selected against two intracellular members of the MAPK pathway: H-Ras and Raf-1 ^46^. Binding variants with high-nanomolar to low-micromolar affinity were either indirectly expressed from encoding constructs or directly transduced using a cell-penetrating peptide (CPP) transfection reagent. With either approach, partial inhibition of tumor necrosis factor-alpha in a synovial cell line induced secretion of the inflammatory mediators interleukin-6 and prostaglandin E _2_^47^. However, inhibition of proliferation was observed only with directly transduced protein and in general produced a greater degree of inhibition of inflammatory mediators than the ectopically expressed variants, suggesting that more protein is delivered via transduction than transfection. Although neither form resulted in potent inhibition, this may be more related to their modest affinities rather than a mechanistic limitation. These examples not only serve as state-of-the-art demonstrations of engineering for intracellular protein targeting but also lay the foundations for future efforts aimed at using engineered proteins in therapeutic applications ( Figure 1).

## Intracellular delivery of proteins

To date, the vast majority of therapeutics targeting intracellular proteins are small molecules, which, owing to their small size and amphiphilic properties, can partition into and pass through cell membranes. In stark contrast, the most daunting challenge to the use of protein-based therapeutics for targeting of intracellular macromolecules is the fact that most proteins do not cross hydrophobic membranes efficiently.

For research, the delivery of proteins intended to modulate the function of intracellular protein activity is most often accomplished by transfection of DNA encoding either native or engineered proteins, which enables endogenous transcriptional and translational machinery to express them ectopically ^48^. Though the delivery of proteins can be effective *in vitro*, challenges with systemic delivery, poor efficiency of delivery, toxicity ^49^, and additional layers of transcriptional and translational regulation make the goal of controlled protein expression for therapeutic application highly challenging. Viruses have been extensively explored for gene therapy, and ways of co-opting their ability to inject genetic cargo inside the cell have been devised for protein delivery ^50^. However, issues associated with viral delivery—including immunogenicity, inflammation and cytotoxicity, a lack of precise control over expression levels (and thus dosing), and complications associated with genetic integration during stable transfection (for example, gene disruption and proto-oncogene activation)—have slowed progress and limited clinical success ** ^51^.

Alternately, novel ways of interrupting protein expression based on RNA interference technologies have also been developed in order to disrupt protein function at the level of transcription by binding to mRNA transcripts and marking them for degradation. Despite their enormous utility, RNA interference techniques suffer from the inability to target post-translational modifications, instability, and off-target effects as well as issues associated with viral delivery as discussed above. Conversely, transfection with mRNA transcripts can bypass transcriptional controls and does not require entry into the nucleus, thus permitting direct expression of encoded proteins even in non-dividing cells. Despite these advantages over transfection with DNA and utility for transient expression of proteins, studies have continued to note variation in expressed protein levels ^52^ that will need to be addressed for realization of its therapeutic potential.

As a result of these limitations, a variety of means of direct introduction of proteins into cells have been explored. Numerous transfection technologies employed for polynucleotides are facilitated by the relative uniformity of their physicochemical properties and overall negative charge arising from their phosphate-sugar backbone. In contrast, proteins lack uniform charge density and possess a broader chemical make-up that makes a single, widely applicable approach to transduction challenging. Accordingly, a number of *in vitro* approaches have emerged for the general aim of ‘profection’ or protein transduction into cells, some of which have only just begun to address the possibility of *in vivo* delivery.

## Physical methods of protein delivery

Electroporation is the transient permeabilization of cells with short bursts of high voltage to allow uptake of biomolecules. Though widely used for DNA delivery, it has also been explored for protein transduction in which uptake and observation of activity are rapid in comparison with indirect expression from DNA ^54, 55^. Electroporation is useful because it avoids endosomal trapping by bypassing the endocytic machinery, but it can be used only on small numbers of cells and may cause excessive cell death or aggregation of the protein to be delivered ^53^. Similar approaches have been explored by using sound ^54^, physical deformation caused by passage through a microfluidic device ^55^, or reversible permeabilization with cholesterol-binding agents ^56^.

Protein-based biosensors or signaling modulators have also been introduced into cells by microinjection, and these highlight the advantages of high transduction efficiencies (even in difficult-to-transduce cells) ^58– 62^, spatial and temporal control (thus circumventing endosomal trapping issues), and precisely controlled dosing ^57^. Novel, photo-sensitive nanocarrier protein complexes coupled with light-based activation techniques are similar to microinjection and may offer increased spatial and temporal control over sites and the frequency of protein delivery ^58^. Nevertheless, all of these techniques are limited to small numbers of cells and are likely difficult to adapt for therapeutic delivery.

## Protein delivery with cationic lipids, liposomes, and polymerosomes

Lipid vesicles and polymeric encapsulation of oligonucleotides are widely used methods for the delivery of DNA and RNA into cells. Similar approaches to protein delivery, though useful for protecting proteins from serum degradation or neutralization, can encounter challenges for encapsulation because of the variable stability and greater chemical and structural diversity of proteins. Conventional liposome preparation methods often employ conditions (that is, solvents, sonication, detergents, and so on) that can lead to protein denaturation and loss of activity ^59^. Alternatively, polycationic lipids, similar to those used for transfection of DNA, have been explored and shown to be effective for intracellular delivery of functionally active proteins *in vitro* using a variety of formulations ^66, 67^. Recent studies using lipid complexes of supercharged anionic GFP-Cre protein and Cas9:single guide RNA complex injected into the inner ear of live mice confirm delivery, functional protein activity, and recombinational events in a restricted *in vivo* milieu ^60^. Though cationic lipid preparations have been used systemically for the experimental delivery of genes *in vivo* ^61^, investigations of the distribution and effective protein transduction for systemically administered polycationic lipid:protein complexes have not, to our knowledge, been reported.

Additional liposome and polymer-based platforms for protein delivery have been described using labile particles that are stable outside the cell but decompose upon exposure to either low pH ^62– 65^ or reducing environments ^66^ in a manner that facilitates protein release and exit from endosomes. These studies confirm the entry, distribution, and functional ability of intact antibodies to block the biological role of target proteins inside cells ^62, 63^. Mechanistic insights into ‘polymerosome’ uptake suggest a role for receptor-mediated uptake via class B scavenger receptors (that is, CD36 and SR-BI/II) rather than direct entry, suggesting a particle structure similar to endogenous scavenger receptor ligands (for example, high- and low-density lipoproteins) ^67^ and that alternate formulations of the polymer may enable targeting of other receptors for uptake. Additional studies showed that polymerosomes conjugated with a low-density lipoprotein receptor-related protein-1 (LRP1) ligand and loaded with IgG mediated both transcytosis across the blood-brain barrier and uptake by cells of the central nervous system when injected intravenously into mice ^68^. Additional studies support the use of receptors that mediate transcytosis for the delivery of antibodies ^69^.

## Protein transduction domains

Numerous studies have been published describing the use of designed CPPs or small protein transduction domains (PTDs) as agents capable of delivering proteins into cells. Although the terms CPP and PTD are often used interchangeably in the literature, we employ the terms in a more strict sense in which peptide-based reagents are referred to as CPPs and autonomously folding domains—for example, CH2-His2 zinc-finger domains—are referred to as PTDs. They are numerous but can be broadly classified as cationic peptides, including human immunodeficiency virus type I (HIV) Tat peptides ^70^, *Drosophila* Antennapedia homeoprotein (Antp 43-58)/penetratin ^71, 72^, poly arginine ^73^, amphipathic peptides such as transportan ^74^, MPG ^75, 76^, MAP ^77^, and Pep-1 ^78^, and hydrophobic peptides ^79^. Various CPPs, including TAT ^80– 82^, rabies virus glycoprotein ^83^, a fibroblast growth factor four-derived peptide ^84^, and annexin-derived peptides ^48^, have been used to investigate the *in vivo* distribution of CPP-tagged cargo proteins. In addition to revealing widespread tissue distribution with no apparent cell-type dependence ^81, 84, 85^, studies appear to confirm sufficient delivery across the blood-brain barrier to achieve a neuroprotective effect in models of brain insult ^80, 82, 83, 86^.

However, initial claims of a temperature- and energy-independent (that is, non-endocytic) mechanism ^71^ were met with skepticism and triggered a deeper investigation of the means of translocation that ultimately revealed previously unrecognized issues with cell-surface adhesion ^87^, artifacts of fixation ^88^, and endosomal trapping ^89^. Though further studies have confirmed the role that one or more endocytic pathways play in uptake (including macropinocytosis and clathrin-, caveolin-, and receptor-mediated uptake) ^89– 92^, in addition to a potential contribution by direct entry ^93^, it is apparent that cytosolic delivery of protein cargo is not guaranteed in all applications and that the mechanism and degree of endosomal release are ongoing challenges that need to be addressed to realize the therapeutic potential of CPPs ^94^. The field also suffers from the general lack of an objective comparison of the transduction efficiency, distribution, and cellular fate of more than one CPP. Though studies of this type would be both informative and helpful in dispelling some of the controversy and confusion, the observed dependence of delivery on factors, including the length and specific properties of the CPP, cargo type, CPP-cargo linkers, and cell type ^95, 96^, complicates a clear and quantitative comparison of transduction efficiencies of various CPPs *in vitro*, and only a handful of studies even attempt such a description ^79, 97– 99^.

In addition, despite the many successes of both natural and designed CPPs to effectively deliver protein cargo, their ability to broadly transduce cells and tissues *in vivo* may actually be an impediment to therapeutic ** applications without additional targeting efforts to ensure delivery to desired tissues. Alternately, novel approaches based on natural mechanisms of tissue specificity and cellular entry may offer solutions to delivering bioactive proteins to target tissues in an *in vivo* setting.

## Virus-like particles

Virus-like particles (VLPs) are self-assembling particles composed of viral capsid proteins that, though lacking the genetic components required for infective virus assembly, nevertheless resemble native viruses and enter cells in a similar fashion ^100– 102^. During self-assembly, their ability to encapsidate a variety of non-viral biomolecules, including DNA ^103, 104^, RNA ^105^, small-molecule drugs ^105^, and proteins ^106, 107^, has piqued substantial interest in their development as drug and vaccine delivery agents ^108^. To this end, VLPs can be engineered to express and display heterologous proteins ^109, 110^ and have been exploited primarily as vaccine carriers for their immunogenic and adjuvant properties and ability to carry cargo into the cell interior ^111, 112^.

The advantages of direct over indirect protein transduction are being recognized, and the potential of VLPs to act as protein transduction agents for the delivery of bioactive proteins to cells is being explored. A growing number of VLP systems have been shown to be amenable to the packaging and intracellular delivery of proteins: polyomavirus ^113^, murine leukemia virus ^114^, hepatitis B virus ^115^, lentivirus ^116, 117^, retrovirus ^118^, Sendai virus ^119^, and Sesbania mosaic plant virus ^109^. Most notably, the delivery of bioactive proteins such as antibodies ^109, 119^, transcription factors ^114, 118^, and enzymes ^110, 116, 117^ has been confirmed both *in vitro* ^110, 114, 116^ ** and *in vivo* ^117^.

**Figure 2.**
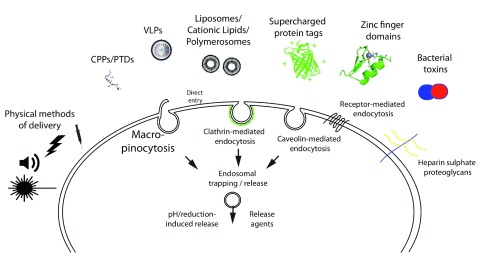
Methods for the direct delivery of proteins into cells. A variety of methods have been described for delivering functional protein to the cell interior and are illustrated along with potential mechanisms of uptake and endosomal release. CPP, cell-penetrating peptide; PTD, protein transduction domain; VLP, virus-like particle.

As therapeutic ** administration requires targeting of diseased cells and tissues *in vivo*, particle distribution is a key consideration in the development of any protein delivery platform. Though some viruses possess natural cell and tissue tropism ^120^, the broad cell specificity exhibited by many of the viruses currently used for delivery would hamper their development as therapeutic agents. It is the opportunity to re-direct or focus broad tropisms for therapeutic advantage that is particularly appealing. Illustrative studies employing different approaches to incorporating antibody scFvs into VLPs have been described by binding to a capsid fusion of the *Staphylococcal aureus* A ** protein Z domain ^121^, incorporation of polyionic fusion peptides with an engineered disulfide bond ^122^, or tagging with the hemagglutinin transmembrane domain ^123^ or a glycosylphosphatidylinositol (GPI) anchor ^124^. Although these studies provide evidence of *in vitro* cell selectivity, a recent study using peptides obtained from selection against surface antigens on human hepatocellular carcinoma cells offers a preliminary example of engineered *in vivo* cell selectivity ^125^ and suggests that this may be a potentially viable approach for tailoring VLPs for therapeutic targeting ( Figure 2).

## Future perspectives

As the field of protein engineering advances the development of novel protein scaffolds targeting an increasingly broad segment of the proteome, techniques for protein delivery into cells will likely progress apace. The pioneering examples of non-antibody scaffolds with the ability to modulate intracellular proteins, signaling outcomes, and cell biology provide increasing confidence that they possess therapeutic value and provide incentive for developing innovative and reliable techniques for intracellular protein delivery both *in vitro* and *in vivo*. Current studies provide proof of principle that these aims are within reach, but few examples provide detailed comparisons of delivery mechanisms or analysis of the distribution and fate of delivered proteins. Despite this, new approaches to delivering proteins to the cell interior continue to be advanced. In addition to novel PTD mimics ^126^, other platforms, including supercharged proteins ^127^, bacterial toxins ^128^, and zinc-finger domains ^129^, have been described. Chemical modifications have also been extensively explored for targeted delivery of protein- and RNA-based drugs and may also offer valuable solutions for *in vivo* targeting ^130, 131^. However, in the absence of comprehensive comparisons of uptake, intracellular distribution, and degradation, it is difficult to assess which approach to intracellular delivery is best. From a pharmacological standpoint, these features (that is, the path, destination(s), and lifetime of a delivered agent) will determine whether a delivered protein drug interacts with its target in its native cellular compartment, for how long it interacts and modifies the activity of the target protein, and how the protein drug is ultimately neutralized or degraded. These are features that undoubtedly underlie the effectiveness of a protein drug whose target is found inside the cell and must be characterized to develop effective formulation and dosing regimens that ensure efficacy and avoid toxicity.

Many of the approaches described thus far have relied on endosomal uptake and may suffer from reduced therapeutic efficacy due to entrapment. Though problematic, efforts toward developing platforms that address this challenge ^62– 65^ as well as toward developing additional means of escape ^34, 132^ may offer viable solutions. Although a preliminary aim of intracellular delivery is to access the cytosol, numerous targets of therapeutic interest are sequestered in other compartments ^133^. Intracellular targeting efforts using signal sequences to localize delivered proteins to subcellular compartments offer potential means of ensuring co-localization of transduced protein drugs and their targets outside of the cytoplasm ^134^. Degradation of proteins delivered by using these approaches will also exert substantial influence over protein activity, lifetime, and cost. With the potential to extend the lifetime of virtually any protein-based therapeutic, novel D protein-binding scaffolds constructed from synthetic D amino acids have been introduced and have the potential to obviate concerns about the susceptibility of natural proteins to proteolytic degradation and immunogenicity ^135, 136^. Furthermore, the small size and high thermostability of many of the non-antibody scaffolds make chemical synthesis and efficient refolding possible, thus reducing cost and avoiding many of the pitfalls associated with production in cellular systems.

These and yet unforeseen approaches and technological innovations will continue to be developed, and it is hoped that they will converge with a primary focus on *in vivo*-targeted delivery of active therapeutic proteins. Consequently, what is now only a lofty goal of using therapeutic proteins inside cells may soon morph into an attainable approach for the treatment of complex pathologies that resist current therapeutic approaches.
